# Effect of dietary fish oil on mouse testosterone level and the distribution of eicosapentaenoic acid-containing phosphatidylcholine in testicular interstitium

**DOI:** 10.1016/j.bbrep.2016.06.014

**Published:** 2016-06-30

**Authors:** Nobuhiro Zaima, Saori Kinoshita, Nao Hieda, Hirona Kugo, Kaori Narisawa, Ayami Yamamoto, Kenichi Yanagimoto, Tatsuya Moriyama

**Affiliations:** a Department of Applied Biological Chemistry, Graduate School of Agricultural Science, Kindai University, 204-3327 Nakamachi, Nara City, Nara 631-8505, Japan; b Human Life Science R&D Center, Nippon Suisan Kaisha, Ltd., Tokyo, Japan

**Keywords:** Matrix-assisted laser desorption ionization mass spectrometry imaging, Testosterone, Fish oil, Eicosapentaenoic acid, Phosphatidylcholine, Testis

## Abstract

Low levels of serum testosterone are characteristically associated with diabetes, coronary atherosclerosis, obstructive sleep apnea, rheumatoid arthritis, and chronic obstructive pulmonary disease. Testosterone replacement therapy is effective against many of these disorders, indicating the importance of maintaining a healthy testosterone level. In this study, we investigated the effects of fish oil on murine testosterone metabolism and analyzed the dynamics of relevant lipids in testes by matrix-assisted laser desorption ionization mass spectrometry imaging. Testosterone was upregulated in mice that received fish oil. In the testicular interstitium, eicosapentaenoic acid-containing phosphatidylcholine was distributed characteristically. These data suggest that eicosapentaenoic acid is involved in testosterone metabolism.

## Highlights

• Testosterone was upregulated in mice that received fish oil.
• In the testicular interstitium, EPA-containing PC was distributed characteristically.
• Leydig cells have a system to incorporate EPA selectively into the plasma membrane.
• EPA may be crucial in testosterone metabolism.

## 1 Introduction

Testosterone is a steroid hormone secreted mainly by Leydig cells in the testicular interstitium of males. It plays an important role in the maintenance of male reproductive tissues (such as testes and prostate), spermatogenesis, and in the promotion of secondary sexual characteristics such as increase in muscle mass [1]. Although testosterone functions in biological regulation, it is also associated with several disorders. The onset and development of several diseases is prevalent in male populations with low testosterone levels. Men with low levels of serum testosterone are at twice the risk of developing diabetes compared to men with normal testosterone level [2]. Phillips et al. reported that low serum testosterone can be a risk factor in developing coronary atherosclerosis [3]. Low serum testosterone levels can also be seen in patients with obstructive sleep apnea, rheumatoid arthritis, and chronic obstructive pulmonary disease [4]. Testosterone replacement therapy is an acknowledged treatment approach for many of these disorders; thus, maintaining healthy testosterone levels can prevent disease onset or disease progression, in some instances [4].

Dietary habits can modulate testosterone metabolism. In humans, daily urinary excretion of testosterone is 13% higher with a high-fat, low-fiber diet than that with a low-fat, high-fiber diet [5]. An earlier study showed that dietary fish oil influences testosterone synthesis and alters fatty acid composition in rat testicular plasma membranes [6]. These reports collectively suggest that fatty acid composition of food affects testosterone metabolism. Nonetheless, it is unclear whether specific fatty acids get incorporated into the testicular interstitium or the fatty acids in the diet directly influence the fatty acid composition of testicular interstitium plasma membranes. The challenge in understanding these aspects is partly attributed to the difficulties in analyzing spatial distribution of specific lipids in tissues. Matrix-assisted laser desorption ionization mass spectrometry imaging (MALDI-MSI) is a suitable method to analyze the characteristic lipid distribution in different tissues including the testes [7], [8], [9], [10], [11], [12]. In this study, we investigated the effect of fish oil on murine testosterone metabolism and subsequently analyzed the dynamics of relevant lipids in mouse testes by MALDI-MSI. The results of this study provide some insights into the relationship between testosterone metabolism and lipid nutrition.

## 2 Materials and methods

### 2.1 Materials

The MALDI matrix 2,5-dihydroxybenzoic acid was procured from Bruker Daltonics (Bremen, Germany). Glass slides (Fisherbrand Superfrost Plus) were purchased from Thermo Fisher Scientific (MA, USA) for an LTQ-XL linear ion trap mass spectrometer (Thermo Fisher Scientific, San Jose, CA, USA). Indium-tin oxide-coated glass slides for a time-of-flight (TOF)/TOF-type instrument (Autoflex) were procured from Bruker Daltonics. Paraformaldehyde was purchased from Nacalai Tesque (Kyoto, Japan). Carboxymethylcellulose sodium salt (low viscosity) was purchased from Sigma-Aldrich Co. (MO, USA). All other reagents used in this study were of the ultrapure grade available.

### 2.2 Animal experiments

All animal experiments were approved by the Institutional Animal Care and Use Committee and were conducted according to the Kindai University Animal Experimentation Regulations (approval number KAAG-25-002). Mice of the ddY strain were provided with food and water *ad libitum* in a humidity-controlled room, in a 12-hour light/12-hour dark cycle. The room temperature was maintained at 25 °C±1 °C. Nutritional supplements were administered via two routes: dietary and gavage. In the dietary administration groups, mice (male, 8 weeks old; Japan SLC, Inc., Hamamatsu, Japan) were randomly subdivided into two groups—control group and dietary fish oil group—and provided with food for 10 weeks (n=5). The mice in the dietary fish oil group were provided food supplemented with fish oil consisting of purified triglycerides extracted from sardines (please mention the concentration; Nippon Suisan Kaisha, Ltd., Tokyo, Japan) during this period. Dietary components and fatty-acid composition are shown in Table 1, Table 2. In gavage groups, mice (male, 15 weeks old; Japan SLC, Inc.) were provided with food (same as the control group in dietary group) for 4 weeks, and subsequently subdivided into two groups—control group and fish oil gavage administration group (n=5). Carboxymethylcellulose was orally administered to the control group for 7 d. Fish oil (Nippon Suisan Kaisha) was orally administered once a day (2285 mg/[kg d]) to the gavage group for 7 d. Body weight and food intake were measured every 3 d.

**Table 1:** Dietary components.

	Control group	Fish oil group
	(g/100 g)	(g/100 g)
Choline chloride	0.2	0.2
Cystine	0.3	0.3
AIN-93 vitamin mix	1	1
AIN-93G mineral mix	3.5	3.5
Cellulose	5	5
Sucrose	10	10
Casein	22.2	22.2
Cornstarch	27.8	27.8
Lard	25	25
Olive oil	5	0
Fish oil	0	5

**Table 2:** Fatty acid composition.

Fatty acid	Olive oil (%)	Fish oil (%)
8:0	–	–
10:0	–	–
12:0	–	–
14:0	–	5.2
16:0	10.8	6.6
16:1	1.0	9.3
16:2	–	1.7
16:3	–	2.8
16:4	–	4.8
18:0	2.7	0.5
18:1	77.1	9.6
18:2 n-6	7.8	1.4
18:3 n-3	0.6	0.9
18:4 n-3	–	5.1
20:4 n-6	–	1.3
20:4 n-3	–	1.1
20:5 n-3	–	30.8
22:5 n-6	–	0.3
22:5 n-3	–	2.9
22:6 n-3	–	15.7

### 2.3 Tissue collection and preparation of tissue section

Blood samples were obtained from the inferior vena cava of mice, under anesthesia. The mice were then perfused through the left cardiac ventricle with an isotonic sodium chloride solution. The collected testes were frozen on a plate cooled with liquid nitrogen without any chemical fixation. Consecutive 10-μm sections were prepared using a cryostat (CRYOCUT CM 1850; Leica Microsystems, Wetzlar, Germany). Successive slices were mounted onto glass slides, and were used for immunohistochemical staining and analyses by means of an LTQ-XL linear ion trap mass spectrometer. Indium-tin oxide-coated glass slides were used for TOF analyses.

### 2.4 Immunohistochemical staining

The slices were fixed with 4% paraformaldehyde for 10 min. After rinsing in phosphate-buffered saline (PBS), endogenous horseradish peroxidase in the tissue slices was blocked using aqueous hydrogen peroxide (a 3% solution in methanol) for 8 min. After rinsing in PBS, the tissue slices were blocked with Blocking One Histo (Nacalai Tesque). The slices were incubated overnight with an antibody against testosterone (1:50 dilution; Cloud-Clone Corp., TX, USA), at 4 °C. On the following day, the slices were rinsed in PBS and incubated with a secondary antibody conjugated with horseradish peroxidase. The slides were developed with diaminobenzidine (Vector Laboratories, Burlingame, CA, USA). Quantitative analysis of the histological staining was performed using the ImageJ software (National Institutes of Health, MD, USA).

### 2.5 MSI

A 50 mg/mL solution of 2,5-dihydroxybenzoic acid in a methanol: water mixture (7:3, v/v) served as a matrix. The matrix solution was sprayed uniformly over the slices by means of an airbrush with a 0.2 mm caliber nozzle (Procon Boy FWA Platinum; Mr. Hobby, Tokyo, Japan). MSI was performed using a MALDI TOF/TOF-type instrument (Autoflex) and the LTQ-XL linear ion trap mass spectrometer. Autoflex was used for imaging based on the MS data, and LTQ-XL was mainly used for imaging based on tandem mass spectrometry (MS/MS) data. This was done because Autoflex is suitable for simultaneous analysis of multiple samples, whereas LTQ-XL can perform simultaneous imaging on the basis of MS and MS/MS data. The data were acquired with a step size of 50 µm in both analyses. The FlexImaging software 4.0 (Bruker Daltonics) and ImageQuest software (Thermo Fisher Scientific) were used to create two-dimensional ion density maps. Normalization of the spectra to the total ion current was also performed in the imaging software. The distribution of the MS/MS product ions was visualized without normalization.

### 2.6 Serum testosterone assay

Serum testosterone was quantified using the Testosterone EIA Kit (Cayman Chemical, MI, USA).

### 2.7 Hematoxylin-Eosin (HE) Staining

Tissue sections were placed in hematoxylin for nuclear staining for 10 min, and then decolorized in acid alcohol (1% HCl in 70% ethanol). After rinsing in tap water, the sections were stained with eosin for 5 min, and then dehydrated in ethanol (80%, 90%, and 100%). Thereafter, the sections were cleared in xylene and covered with a lipid-soluble mounting medium, Entellan® New (Merck KGaA, Germany), and glass cover slips.

### 2.8 Statistical analysis

The statistical significance was determined by two-sided Student's *t* test. P-values <0.05 were considered significantly different. The calculations were performed in the Stat View 5.0 software (SAS Institute, Tokyo, Japan).

## 3 Results

### 3.1 Testosterone levels in testes and serum

**Fig. 1:**
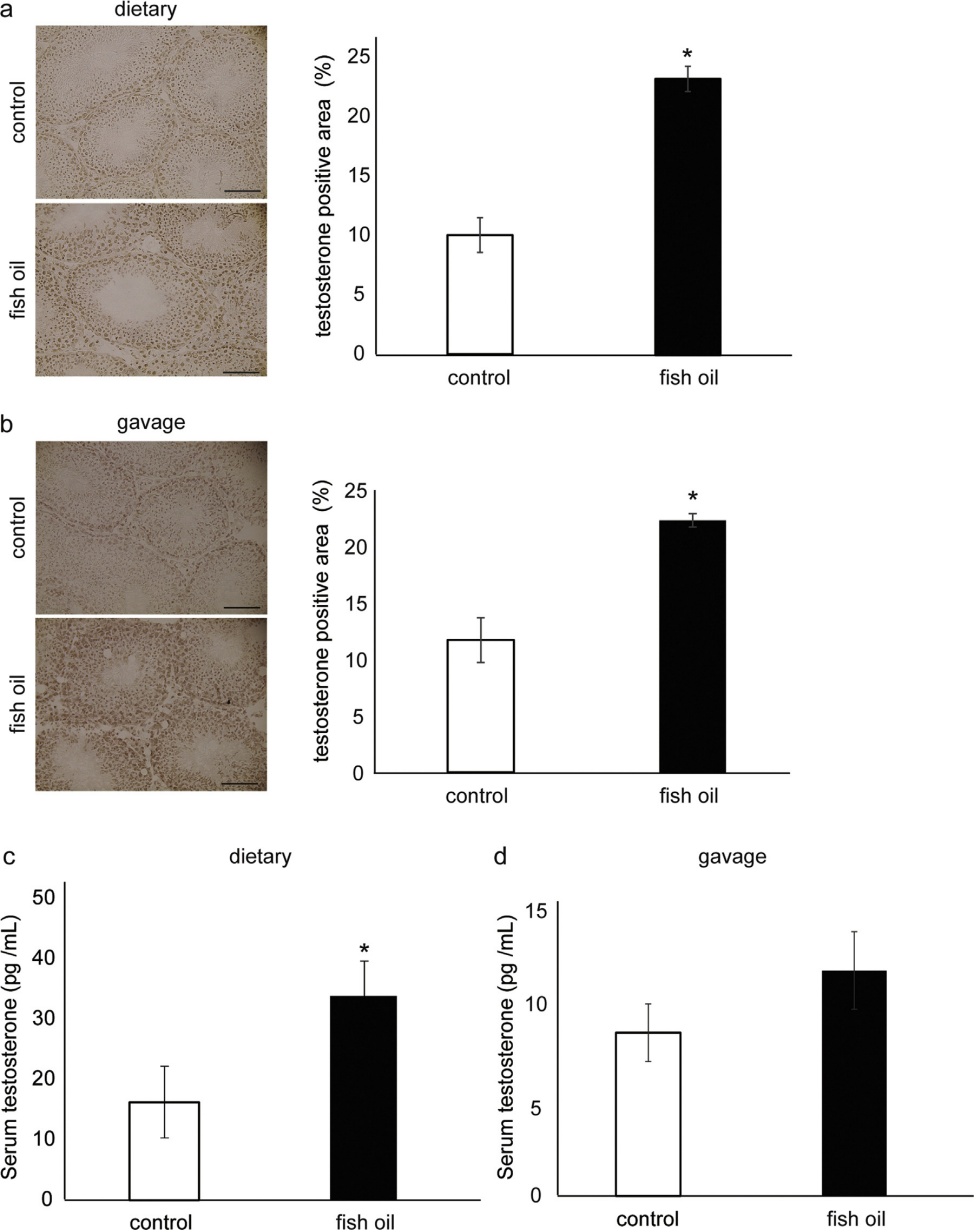
Testosterone levels in dietary and gavage administration conditions in relation to fish oil. Immunohistological staining of anti-testosterone in the dietary administration group and the testosterone-positive area of sections of a testis (a). Scale bars=250 µm. Immunohistological staining of anti-testosterone in the gavage group and the testosterone-positive area of sections of a testis (b). Scale bars=250 µm. The serum testosterone level in the dietary administration group (c). The serum testosterone level in the gavage group (d). The data are presented as mean±SEM, n=5;*P<0.05 compared to the control group.

The effect of fish oil on testosterone metabolism in mice has not been reported thus far. We studied the testosterone levels in mice under two experimental conditions: dietary administration and gavage. The positive area of testosterone in the testes in the dietary fish oil group was significantly higher than that in the control group (Fig. 1a). Similarly, the positive area of testosterone in testes of mice from the fish oil gavage group was significantly higher than that in the control group (Fig. 1b). Serum testosterone in dietary fish oil group was significantly higher than that of the control group (Fig. 1c). Serum testosterone in the fish oil gavage group also showed an increasing trend in comparison to the control group (Fig. 1d). Food intake, body weight, and weight of the testes were not significantly different between the two groups (data not shown).

### 3.2 Phosphatidylcholine (PC) composition of testes

We analyzed the proportions of major PCs in testes. The number of carbon atoms and double bonds in the fatty acid moiety of PC are shown as an integrated value of two fatty acids in *sn*-1 and *sn*-2 positions (Table 3). The proportions of PC(34:1)-*m/z* 798, PC(36:5)-*m/z* 818, PC(36:2)-*m/z* 824, PC(36:1)-*m/z* 826, and PC(38:6)-*m/z* 844 in the testes of mice in the dietary fish oil group were significantly higher than those in the control group. The proportions of PC (36:4)-*m/z* 820, PC(38:5)-*m/z* 846, PC(38:4)-*m/z* 848, and PC(40:7)-*m/z* 870 in the testes of mice in the dietary fish oil group were significantly lower than those in the control group were. The proportion of PC(36:5)-*m/z* 818 in the testes of mice in the fish oil gavage group was significantly higher than that in the control group, whereas the proportion of PC(40:6)-*m/z* 872 was significantly lower than that of control group.

**Table 3:** Phosphatidylcholine composition of testis in dietary and gavage group.

		Dietary administration		Gavage administration	
*m/z* value	Assignment	Control	Fish oil	Control	Fish oil
*m/z* 796	PC(34:2)+K	3.21±0.12	3.37±0.11	2.72±0.13	2.64±0.13
*m/z* 798	PC(34:1)+K	18.81±0.35	20.69±0.52*	18.45±0.45	19.96±0.66
*m/z* 800	PC(34:0)+K	5.80±0.14	6.03±0.15	5.94±0.17	6.29±0.19
*m/z* 818	PC(36:5)+K	1.04±0.04	1.31±0.01*	1.32±0.05	1.58±0.06*
*m/z* 820	PC(36:4)+K	17.28±0.63	14.80±0.40*	15.56±0.40	14.66±0.30
*m/z* 824	PC(36:2)+K	4.20±0.09	4.89±0.19*	4.75±0.09	4.53±0.09
*m/z* 826	PC(36:1)+K	4.99±0.17	5.97±0.16*	4.77±0.31	5.17±0.15
*m/z* 844	PC(38:6)+K	7.53±0.17	12.25±0.35*	8.17±0.37	8.84±0.30
*m/z* 846	PC(38:5)+K	18.58±0.78	16.22±0.39*	19.99±0.58	19.87±0.32
*m/z* 848	PC(38:4)+K	11.08±0.69	8.36±0.24*	9.83±0.23	8.89±0.48
*m/z* 870	PC(40:7)+K	3.55±0.21	2.92±0.15*	4.39±0.19	3.88±0.11
*m/z* 872	PC(40:6)+K	3.94±0.30	3.19±0.14	4.11±0.13	3.68±0.06*

Values are expressed as the means±SE (n=5).

The number of carbon atoms and double bonds in the fatty-acid moiety of PC are shown as an integrated value of two fatty acids in sn-1 and sn-2 positions.

* represents a significant difference (p<0.05).

### 3.3 MALDI-MSI analyses of testes

**Fig. 2:**
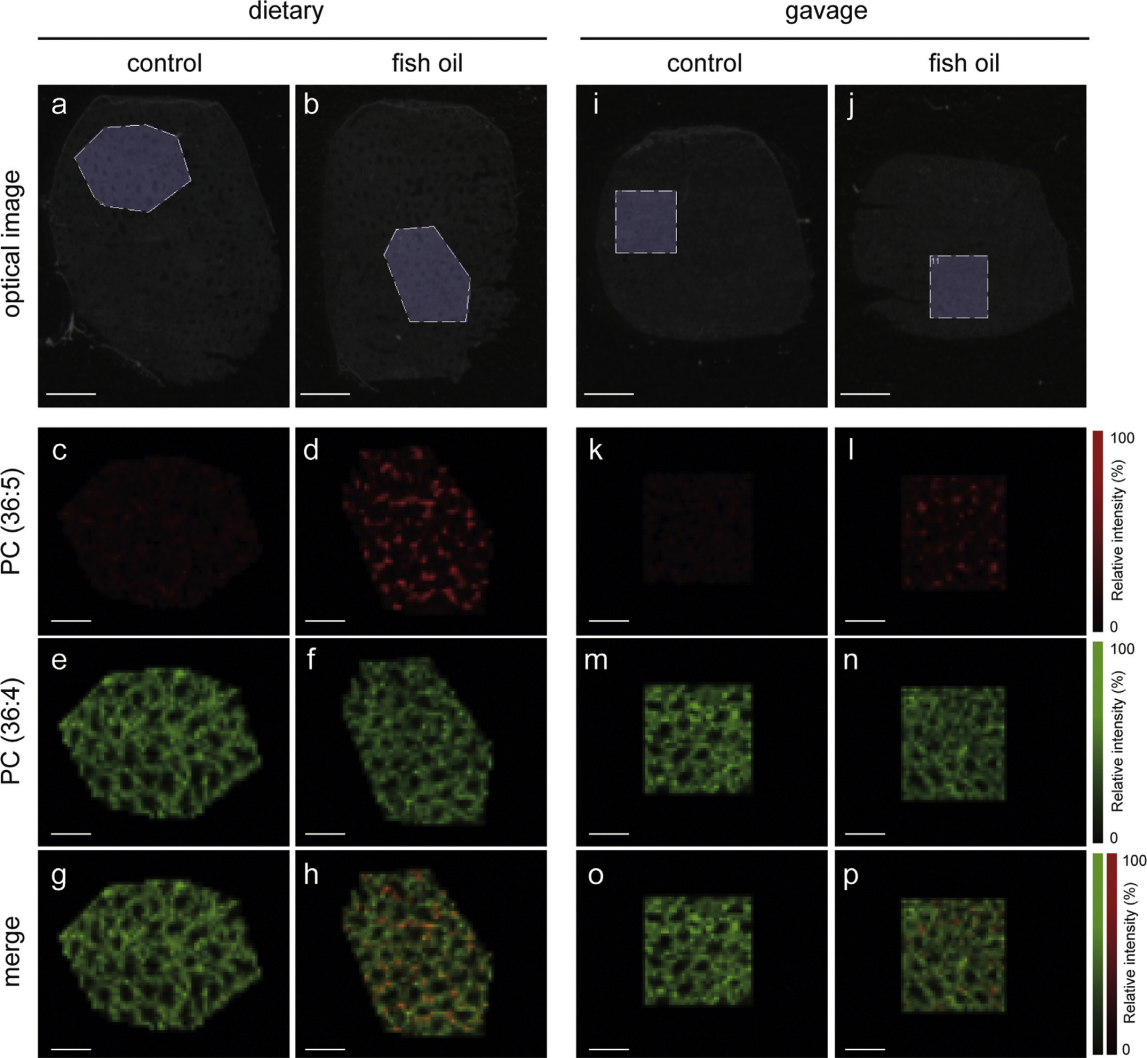
Distribution of phosphatidylcholine (PC) molecular species in murine testes. Optical images of a testis of a mouse from the dietary administration group (a and b). Regions of matrix-assisted laser desorption ionization mass spectrometry imaging (MALDI-MSI) analysis are highlighted. Distributions of PC(36:5) and PC(36:4) (c–f). A merged image of PC(36:5) and PC(36:4) (g and m). Optical images of a testis of a mouse from the gavage group (i and j). Regions of MALDI-MSI analysis are highlighted. Distributions of PC(36:5) and PC(36:4) (k–n). A merged image of the distributions of PC(36:5) and PC(36:4) (o and p); n=5. Scale bars=500 µm (optical image) and 1000 µm (MALDI-MSI data).

Since the increase in PC(36:5) level in the testes was common to both experimental groups (dietary and gavage), the distribution of PC(36:5) was analyzed by MALDI-MSI (Fig. 2). Optical images of a testis and measurement area for the dietary administration group are shown in Fig. 2a and b. The characteristic distribution of PC(36:5) is illustrated in Fig. 2c and d. The distribution of PC(36:5) did not correspond to PC(36:4), which is a marker of seminiferous tubules, [12] (Fig. 2e–h). The same distribution pattern was observed in the gavage group (Fig. 2i–p).

### 3.4 Distribution of PC(36:5) in testes

**Fig. 3:**
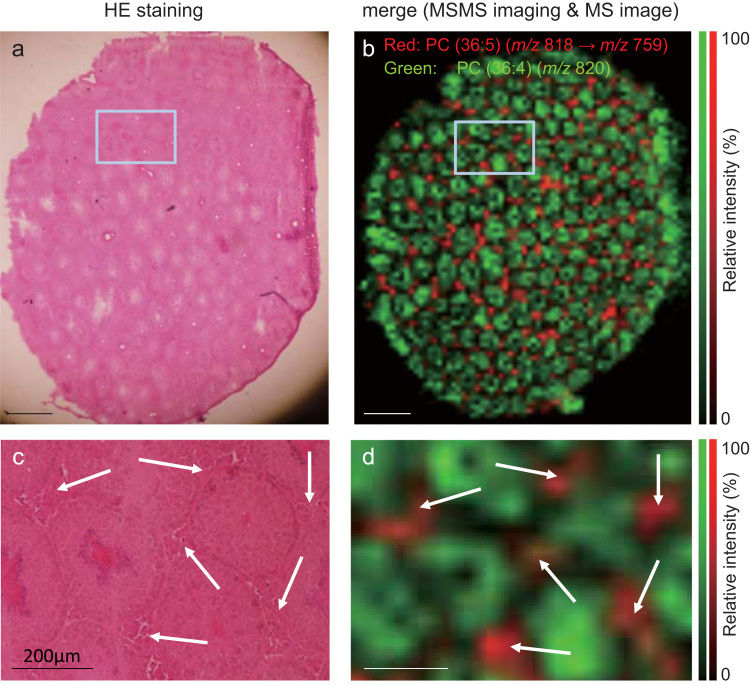
Comparison of the phosphatidylcholine PC(36:5) data by using hematoxylin-eosin (H&E) staining. The latter staining of a testis from a mouse fed fish oil (a). A merged image of the distributions of PC(36:5) and PC(36:4) (b). Scale bars=1000 µm. A high-power magnified field of the square region from panel a (c). Arrows indicate the testicular interstitium. A high-power magnified field of the square region from panel b (d). Arrows show the testicular interstitium; n=3. Scale bars=200 µm.

The observed distribution of PC(36:5) was characteristic; this distribution was compared with the H&E staining of consecutive slices (Fig. 3a). To enhance the accuracy of data on PC distribution, MS/MS imaging of *m/z* 818 was performed. The distribution of *m/z* 759 produced from *m/z* 818 was visualized as the distribution of PC(36:5) because a neutral loss of 59 was indicative of a PC (Fig. 3b). No reliable product ions were observed in the MS/MS spectrum that corresponded to the fatty acid tails of PC, under the experimental conditions. Representative MS/MS spectrum is shown in Supplemental figure 1. According to the H&E staining and MS/MS imaging data, PC(36:5) was localized to the testicular interstitium (Fig. 3c and d).

### 3.5 PC composition of the testicular interstitium

We also studied PC composition of the testicular interstitium because PC(36:5) was distributed characteristically in the testicular interstitium (Table 4, Table 5). The proportions of PC(34:2)-*m/z* 796, PC(34:0)-*m/z* 800, PC(36:5)-*m/z* 818, and PC(38:6)-*m/z* 844 in the testicular interstitium of mice in the dietary fish oil group were significantly higher than those in the control group (Table 4), whereas the proportion of PC(38:4)-*m/z* 848 was significantly lower (Table 4). The proportion of PC(36:5)-*m/z* 818 in the testicular interstitium of mice in the fish oil gavage group was significantly higher than that in the control group (Table 5), whereas the proportion of PC(36:4)-*m/z* 820 was significantly lower (Table 5). The proportion of PC(36:5)-*m/z* 818 in seminiferous tubules of mice in the fish oil gavage group was higher than that in the control group (Table 4). The proportion of PC(36:5)-*m/z* 818 in seminiferous tubules of mice in the dietary fish oil group was not significantly different from that in the control group (Table 5).

**Table 4:** Phosphatidylcholine composition of testis in dietary group.

		Testicular interstitium		Seminiferous tubule	
*m/z* value	Assignment	Control	Fish oil	Control	Fish oil
*m/z* 796	PC(34:2)+K	2.68±0.25	3.43±0.14*	3.05±0.31	2.95±0.22
*m/z* 798	PC(34:1)+K	13.52±1.51	16.45±0.78	13.93±0.62	17.06±0.96*
*m/z* 800	PC(34:0)+K	3.89±0.27	4.78±0.19*	5.01±0.20	5.44±0.19
*m/z* 818	PC(36:5)+K	0.93±0.08	2.02±0.16*	1.07±0.02	1.12±0.07
*m/z* 820	PC(36:4)+K	13.12±0.86	11.27±0.95	11.28±1.52	11.28±0.92
*m/z* 824	PC(36:2)+K	8.00±0.45	9.30±0.73	3.27±0.20	3.50±0.10
*m/z* 826	PC(36:1)+K	14.91±1.14	14.90±1.16	2.79±0.18	3.68±0.19*
*m/z* 844	PC(38:6)+K	4.79±0.47	6.27±0.37*	11.84±1.00	18.69±0.76*
*m/z* 846	PC(38:5)+K	9.07±0.86	10.65±0.59	27.37±0.94	21.84±0.67*
*m/z* 848	PC(38:4)+K	20.95±1.81	14.21±1.20*	9.74±0.33	7.05±0.23*
*m/z* 870	PC(40:7)+K	2.78±0.05	2.64±0.16	6.32±0.60	4.11±0.33*
*m/z* 872	PC(40:6)+K	5.36±0.62	4.09±0.19	4.33±0.37	3.29±0.22*

Values are expressed as the means±SE (n=5).

The number of carbon atoms and double bonds in the fatty-acid moiety of PC are shown as an integrated value of two fatty acids in sn-1 and sn-2 positions.

* represents a significant difference (p<0.05).

**Table 5:** Phosphatidylcholine composition of testis in gavage group.

		Testicular interstitium		Seminiferous tubule	
*m/z* value	Assignment	Control	Fish oil	Control	Fish oil
*m/z* 796	PC(34:2)+K	2.69±0.17	2.99±0.44	2.53±0.13	2.48±0.17
*m/z* 798	PC(34:1)+K	16.84±1.01	17.26±1.29	15.98±1.17	18.09±1.53
*m/z* 800	PC(34:0)+K	5.46±0.30	5.37±0.22	5.65±0.37	6.19±0.41
*m/z* 818	PC(36:5)+K	1.45±0.11	2.49±0.21*	1.06±0.02	1.33±0.04*
*m/z* 820	PC(36:4)+K	15.81±1.45	11.18±0.70*	13.77±1.19	12.31±0.85
*m/z* 824	PC(36:2)+K	7.32±0.66	10.27±0.89	4.06±0.27	3.75±0.26
*m/z* 826	PC(36:1)+K	10.58±1.52	13.79±2.37	2.38±0.17	3.26±0.62
*m/z* 844	PC(38:6)+K	5.39±0.27	5.36±0.50	10.13±0.89	11.16±0.99
*m/z* 846	PC(38:5)+K	11.86±0.70	12.54±0.73	24.26±1.25	23.32±2.10
*m/z* 848	PC(38:4)+K	14.99±1.16	12.08±1.23	8.07±0.44	8.67±0.36
*m/z* 870	PC(40:7)+K	3.13±0.14	3.28±0.21	7.25±0.59	5.55±0.53
*m/z* 872	PC(40:6)+K	4.49±0.28	3.38±0.40	4.84±0.26	3.89±0.11*

Values are expressed as the means±SE (n=5).

The number of carbon atoms and double bonds in the fatty-acid moiety of PC are shown as an integrated value of two fatty acids in sn-1 and sn-2 positions.

* represents a significant difference (p<0.05).

## 4 Discussion

In this study, we studied the effects of fish oil on testosterone metabolism in mice (Fig. 1) and subsequently measured the dynamics of PC molecular species in the testes of mice that received fish oil under the two experimental conditions (Table 3, Table 4). The effect of fish oil administration on testosterone metabolism in this study was consistent with an earlier study in rats that were fed fish oil for 28 d [6]. Among the PC molecular species, the proportion of PC(36:5) significantly increased in the testicular interstitium of mice with both, the dietary and gavage, administration of fish oil (Table 4, Table 5). MALDI-MSI analysis revealed that in the testicular interstitium of mice that received fish oil, PC(36:5) was localized characteristically (Fig. 2, Fig. 3). Considering the abundance of fatty acids in the administered fish oil, the major fatty acid moieties of PC(36:5) can be assigned to 16:0 (palmitic acid) and 20:5 (eicosapentaenoic acid, EPA). These data suggest that EPA is incorporated into the plasma membrane of testicular-interstitium cells in a characteristic manner.

Several regulatory mechanisms are responsible for testosterone synthesis in Leydig cells: (i) regulation of the expression level of luteinizing hormone (LH) receptor in the plasma membrane or its signal transduction pathway; (ii) availability of a precursor of testosterone, such as cholesterol, from blood or synthesis *de novo*; (iii) regulation of de-esterification of stored cholesterol ester for initiation of testosterone synthesis; (iv) regulation of cholesterol transport into mitochondria via a multiprotein complex including such proteins as A-kinase anchoring proteins, steroidogenesis acute regulatory protein, and voltage-dependent anion channel; (v) maintenance of appropriate organelle structure including lipid composition of the plasma membrane; (vi) regulation of expression of steroidogenic enzymes such as CYP11A1, CYP17, 3β-hydroxysteroid dehydrogenase, and 17β-hydroxysteroid dehydrogenase; and (vii) regulation of the cofactors necessary for steroidogenic enzyme action [13]. Sebokova et al. reported that LH-stimulated testosterone synthesis in Leydig cells in rats fed fish oil increased in comparison to rats fed linseed oil [6]. Although the effects of fish oil on this testosterone synthesis pathway remain unknown, the EPA-containing PC may be involved in the testosterone synthesis due to LH-mediated modulation.

The modulation of serum testosterone by a functional food (or similar factors) may be effective in preventing some of the diseases associated with low serum testosterone levels. In this study, fish oil was administered through diet and gavage. Testicular testosterone increased in both experimental conditions but serum testosterone increased only in the dietary administration group (Fig. 1). It has been suggested that long-term fish oil administration can modify the testosterone metabolism at blood level. In this study, we focused on the dynamics of PC in testicular interstitium because PC plays an essential role in synthesizing testosterone and the characteristic distribution of PC (36:5) was observed in the testicular interstitium. However, the characteristic PC dynamics were observed in the seminiferous tubule as well. Administration of fish oil might influence the metabolism of seminiferous tubule. Further studies are needed to understand the effect of fish oil on metabolism of testis.

Although the fish oil that was used in this study contains large amounts of docosahexaenoic acid (DHA; Table 2), the proportion of the DHA-containing PC(40:6) (estimated fatty acid composition 18:0 and 22:6) or PC(40:7) (estimated fatty acid composition 18:1 and 22:6) was not changed in the testicular interstitium of the mice in the both fish oil group (Table 4, Table 5). These data indicate that Leydig cells have an unidentified system to incorporate EPA selectively into the plasma membrane, and EPA may be crucial in testosterone metabolism.

In conclusion, we demonstrated the effects of fish oil on testosterone metabolism in mice and the characteristic distribution of the EPA-containing PC in the testicular interstitium. Our data suggest that organ-specific tissues have a system or multiple systems to selectively incorporate certain lipids necessary for tissue functioning. It is possible that differences in the distribution of nutritional components in a tissue influence individual differences in the metabolic response to the diet. It may be worthwhile to consider localization of a food ingredient in a target tissue and to custom-design a dietary ingredient for favorable functioning of tissue-specific metabolism to achieve positive health outcomes.

## Appendix A Supplementary material

Supplementary material: Supplemental Fig. 1 MS/MS spectrum of *m/z* 818.

## Footnotes

Appendix A Supplementary data associated with this article can be found in the online version at 10.1016/j.bbrep.2016.06.014.
