# Supplementary figures and images for: Effect of dietary fish oil on mouse testosterone level and the distribution of eicosapentaenoic acid-containing phosphatidylcholine in testicular interstitium
Source: Biochem Biophys Rep. 2016 Jun 30;7:259–65. doi: 10.1016/j.bbrep.2016.06.014 (PMC5613343; doi:10.1016/j.bbrep.2016.06.014)

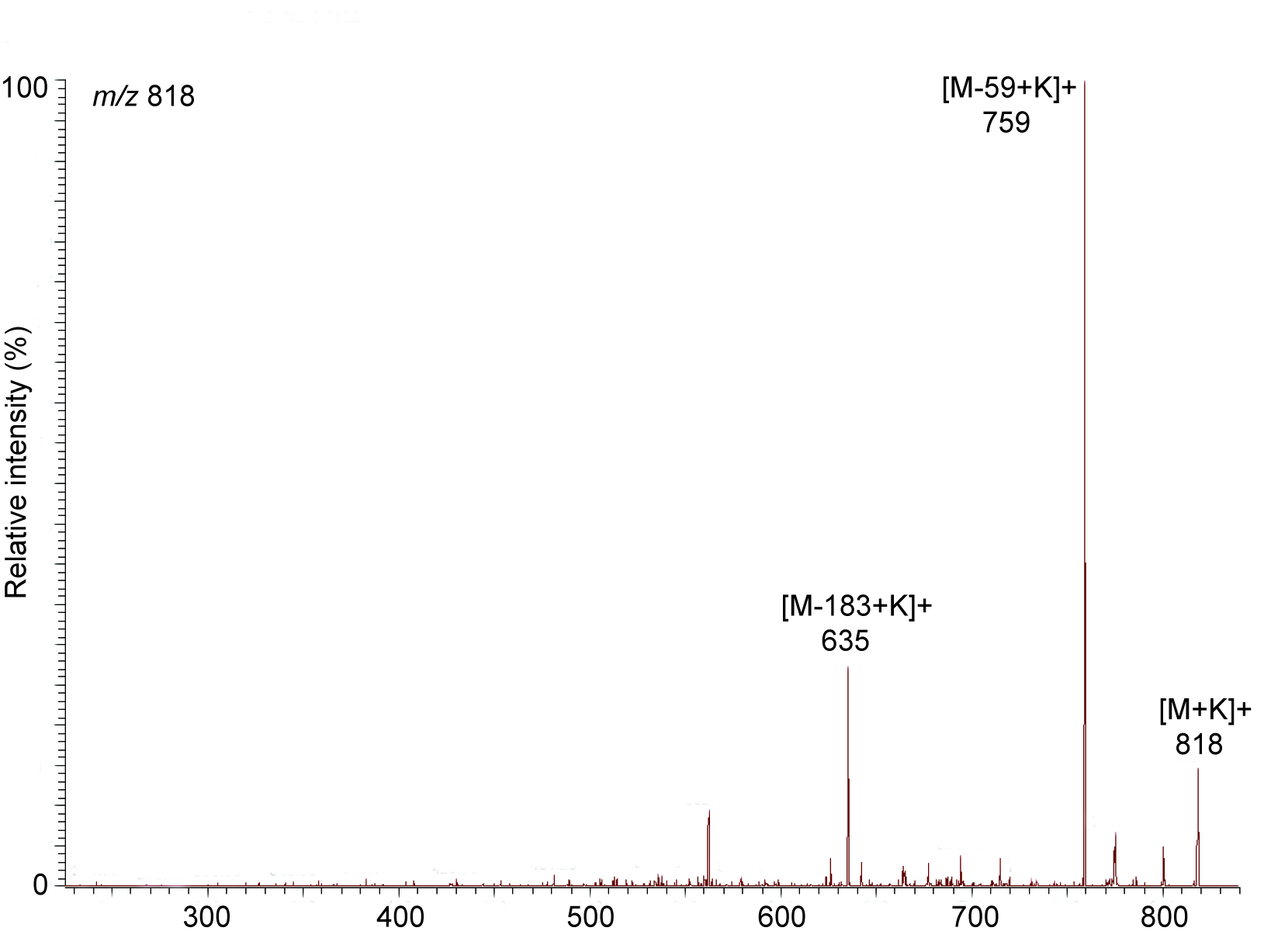

Supplement: Supplementary file 1 — Supplementary material: Supplemental Fig. 1 MS/MS spectrum of m/z 818. [file mmc1.zip › Supplemental Fig1.tif]
