# Supplementary material for: Effect of dietary fish oil on mouse testosterone level and the distribution of eicosapentaenoic acid-containing phosphatidylcholine in testicular interstitium
Source: Biochem Biophys Rep. 2016 Jun 30;7:259–65. doi: 10.1016/j.bbrep.2016.06.014 (PMC5613343; doi:10.1016/j.bbrep.2016.06.014)
Supplement: Supplementary file 2 — Supplementary material [file mmc2.doc]

Conflict of Interest

There is no conflict of interest in this study.
